# Epigenetic impact of a 1-week intensive multimodal group program for adolescents with multiple adverse childhood experiences

**DOI:** 10.1038/s41598-022-21246-9

**Published:** 2022-10-20

**Authors:** Perla Kaliman, Marta Cosín-Tomás, Andy Madrid, Susana Roque López, Elkin Llanez-Anaya, Ligia A. Papale, Reid S. Alisch, Richard J. Davidson

**Affiliations:** 1 Center for Healthy Minds, University of Wisconsin-Madison, 625 W. Washington Ave., Madison, WI 53703 USA; 2 Universitat Oberta de Catalonia (UOC), Rambla del Poblenou, 156, 08018 Barcelona, Spain; 3 ISGlobal, Barcelona, Catalonia, Spain; 4 Universitat Pompeu Fabra, Barcelona, Catalonia, Spain; 5 Centro de Investigación Biomédica en red en Epidemiología y Salud Pública (Ciberesp), Madrid, Spain; 6 Department of Neurological Surgery, University of Wisconsin-Madison School of Medicine and Public Health, Madison, WI 53792 USA; 7 Association Innocence in Danger Colombia (IIDC), 33 Avenue Saint Charles, 34090 Montpellier, France; 8 Masira Institute, University of Santander (UDES), Bucaramanga, Colombia

**Keywords:** Psychology, Biomarkers, Health care

## Abstract

Adverse childhood experiences (ACEs, i.e., abuse, neglect, household dysfunction) represent a potential risk factor for a wide range of long-lasting diseases and shorter life expectancy. We recently described a 1-week residential group program, based on mindfulness training, artistic expression and EMDR group therapy, that significantly reduced PTSD-related symptoms and increased attention/awareness-related outcomes in adolescent girls with multiple ACEs in a randomized controlled study. Since epigenetic mechanisms (i.e., DNA methylation) have been associated with the long-lasting effects of ACEs, the present report extends these prior findings by exploring genome-wide DNA methylation changes following the program. Saliva samples from all participants (n = 44) were collected and genomic DNA was extracted prior (T1) and following (T2) the intervention. Genome-wide DNA methylation analysis using the MethylationEPIC beadchip array (Illumina) revealed 49 differentially methylated loci (DML; *p* value < 0.001; methylation change > 10%) that were annotated to genes with roles in biological processes linked to early childhood adversity (i.e., neural, immune, and endocrine pathways, cancer and cardiovascular disease). DNA sequences flanking these DML showed significant enrichment of transcription factor binding sites involved in inflammation, cancer, cardiovascular disease, and brain development. Methylation changes in SIRT5 and TRAPPC2L genes showed associations with changes in trauma-related psychological measures. Results presented here suggest that this multimodal group program for adolescents with multiple victimization modulates the DNA methylome at sites of potential relevance for health and behavioral disorders associated with ACEs.

## Introduction

The exposure to chronic and severe negative life experiences during early childhood is associated with the development of a host of physical and mental health problems later in life^1^. Adverse childhood experiences (ACEs) include physical, sexual and verbal abuse, physical and emotional neglect, witnessing violence at home, a family member suffering from addictions, mental health issues or incarcerated, and losing a parent to separation, divorce or other reason^2^. Children who have experienced four or more ACEs are more likely to develop long lasting health issues such as diabetes, heart disease, overweight or obesity, cancer, respiratory disease, mental health conditions, alcohol and drug abuse, interpersonal and self-directed violence and sexual risk taking^3^.

There is growing evidence suggesting that epigenetic modulation is one of the molecular mechanisms through which stressors interact with the genome. Epigenetic information regulates gene expression and, although relatively stable, the epigenetic landscape is highly sensitive to environmental exposures^4,5^. DNA methylation is one of the most widely studied epigenetic modifications in which a methyl group is added to a cytosine residue, most commonly in the context of cytosine-guanine dinucleotides (CpG). Children exposed to severe adversity show DNA methylation changes in genes involved in the vulnerability to stress, neurotransmission, inflammatory responses and behavior^6–10^. Negative childhood exposures can trigger DNA methylation changes in genes that modulate anxiety and related phenotypes, such as the oxytocin receptor, glucocorticoid receptor, serotonin transporter gene, brain-derived neurotrophic factor and glutamate receptor^11–15^. Early-life maternal and paternal stressors are predictive of DNA methylation changes detected in adolescents^16^ and both ACEs and DNA methylation changes at the glucocorticoid receptor gene have been associated with increased risk of psychopathologies during adolescence^17^. Moreover, adverse experiences have been associated to an accelerated biological aging^18^. The deviation between the DNA methylation age and the chronological age is a measure of the epigenetic aging rate^19,20^. In children, the Pediatric-Buccal-Epigenetic (PedBE) clock is a tool to measure the biological age, providing an understanding of the environmental exposures that might influence child health and disease^21^. Recent findings show that psychologically adverse or violent home environments can accelerate epigenetic aging in youth^22^. Similarly, neighborhood violence or elevated parental depressive symptoms have been associated with both emotional distress and accelerated epigenetic aging in children^18,23–25^. Importantly, an accelerated rate of epigenetic aging predicts the risk of many chronic conditions such as obesity, cancer, Alzheimer's disease, cardiovascular disease, and all-cause mortality risk^26^.

Recent research shows that positive childhood experiences predict positive outcomes in long-term health and can also neutralize the negative impact of ACEs on adult health^27^. In this context, interventions to increase awareness and understanding of childhood adversities and to promote family connection have been proposed as strategies to influence health and well-being later in life^28–30^. In addition, multimodal programs that combine several approaches such as cognitive behavioral therapy, exercise, yoga, music, art, EMDR (Eye Movement Desensitization and Reprocessing) therapy, individual counselling and interactions with animals have been proposed to improve wellbeing and mental health in child victims of multiple ACEs^31,32^. Notably, in rodents, an enriched environmental model, which includes cognitive, somatosensorial, motor and visual stimulation, reduces the negative psychological and behavioral consequences of early adversity by modulating trauma-related epigenetic marks and improving neurogenesis and synaptic plasticity^33–35^.

We recently described the protocol and mental health impact of a 1-week multimodal intervention group (n = 44 girls) program for adolescents (aged 13–16 years) reporting 4 or more ACEs^36^. After completing the program, the intervention group showed significant reduction in trauma-related outcomes (− 73% in the Short PTSD Rating Interview (SPRINT) scale; − 26% in the Child PTSD Symptom Scale (CPSS)) and a 57% improvement in attention/awareness-related outcomes Mindful Attention Awareness Scale-Adolescents (MAAS-A). This program addresses trauma through evidence-based therapeutic approaches, in an enriched environment that provides social, somatosensory and cognitive stimulation. Based on the literature discussed above, we hypothesize that these conditions may trigger DNA methylation changes in genes involved in the pathophysiology of multiple ACEs, such as vulnerability to stress, neurotransmission, inflammatory responses, behavior and cell aging. We hypothesize that some of the DNA methylation changes may correlate with the mental health improvements that we have previously reported in the same sample^36^, providing insights for future mechanistic research. To start testing this hypothesis, we profiled genome-wide DNA methylation levels in saliva samples from control and intervention group participants, at baseline (T1) and post-intervention (T2), in order to detect potential physiologically relevant DNA methylation changes.

## Results

### Intensive multimodal 1-week group program causes genome-wide alterations in DNA methylation

To identify the impact of the intervention on DNA methylation levels at each CpG on the Human MethylationEPIC array (*N* =  > 850,000 sites), we used an ANCOVA model adjusting for DNA methylation level at baseline (T1), BMI, age, ACEs score and cell type proportions (see details in “Methods”). This approach revealed that 49 DML exhibited a *p* value < 0.001 and a change in DNA methylation level greater than 10% (Table 1), while 195 DML showed a *p* value < 0.001 and a change in DNA methylation level greater than 5% (Supplementary Table S1).

**Table 1:** Intervention-sensitive differentially methylated loci (DML) with p-value lower than 0.001 and a DNA mean difference (T2–T1) of 10% or more (n = 49).

CpG ID	Chromosome	Position	Strand	Relative to island position	FDR	*P* value	Mean difference (t2−t1)	UCSC reference gene symbol	UCSC reference gene name	UCSC reference gene group	UniProt function
cg18252633	chr11	73054401	–	S_Shore	0.41	8.25E−07	− 0.10	*ARHGEF17*	Rho guanine nucleotide exchange factor 17	Body	Acts as guanine nucleotide exchange factor (GEF) for RhoA GTPases, involved in actine cytoskeleton organization
cg11761483	chr17	70723386	–	OpenSea	0.65	5.85E−06	0.15	*SLC39A11*	Solute carrier family 39 member 11	Body	Functions as a zinc ion transmembrane transporter
cg11377646	chr1	11455041	+	OpenSea	0.69	1.45E−05	0.11	*–*	–	–	–
cg00537196	chr14	52688271	–	OpenSea	0.49	2.67E−05	0.14	*–*	–	–	–
cg16270222	chr17	41446396	–	Island	0.65	5.03E−05	0.10	*–*	–	–	–
cg08827579	chr9	117150458	+	OpenSea	0.69	5.20E−05	0.15	*AKNA*	AT-Hook transcription factor	5'UTR	Centrosomal protein that plays a key role in cell delamination by regulating microtubule organization; involved in regulation of transcription and inflammatory responses
cg05105832	chr10	64520254	–	OpenSea	0.66	5.57E−05	0.13	*–*	–	–	–
cg21052873	chr12	124938573	+	N_Shelf	0.76	7.37E−05	0.10	*NCOR2*	Nuclear receptor corepressor 2	Body	Transcriptional corepressor, involved in the regulation of several siglnaling pathways such as Notch
cg20497635	chr17	998504	+	OpenSea	0.76	1.14E−04	0.17	*ABR*	Active BCR-related gene	Body	Functions as an important regulator of RAC1 activity in neurons and macrophages (regulating synaptic transmission, and GTPase mediated signal transduction)
cg02202133	chr9	126312322	–	OpenSea	0.76	1.20E−04	− 0.12	*DENND1A*	DENN domain containing 1A	Body	Guanine nucleotide exchange factor (GEF) that regulates clathrin-mediated endocytosis of synaptic vesicles and mediates exit from early endosomes
cg16306870	chr3	194868790	+	OpenSea	0.79	1.93E−04	0.18	*XXYLT1; C3orf21*	Xyloside xylosyltransferase 1	Body	Glycosyltransferase targetting Notch proteins and coagulation factors, among others
cg03208742	chr12	124475432	–	OpenSea	0.69	1.95E−04	− 0.10	*ZNF664;ZNF664-FAM101A*	Zinc finger protein 664	5'UTR	Zing finger protein involved in transcriptional regulation
cg25735425	chr17	40307262	–	Island	0.79	2.32E−04	0.11	*RAB5C*	Ras-related protein Rab-5C	TSS1500	GTP-binding protein involved in protein transport and vesicular traffic
cg17418085	chr1	31229122	–	OpenSea	0.79	2.36E−04	0.15	*LAPTM5*	Lysosomal-associated transmembrane protein 5	Body	Transmembrane receptor associated with lysosomes; involved in embryogenesis and in adult hematopoiesis
cg01569346	chr6	32064148	+	Island	0.80	3.42E−04	− 0.19	*TNXB*	Tenascin-X	Body	Involved in cell adhesion, mediates interactions between cells and the extracellular matrix
cg07069368	chr6	45294931	–	OpenSea	0.65	3.44E−04	0.20	*RUNX2;SUPT3H*	Runt-related transcription factor 2	TSS1500;5'UTR;Body	Transcription factor involved in osteoblastic differentiation and skeletal morphogenesis
cg05884705	chr15	40600099	+	OpenSea	0.79	3.52E−04	0.17	*PLCB2*	Phospholipase C Beta 2	1stExon;5'UTR	Phosphodiesterase involved in lipid metabolism and signal transduction
cg21005774	chr14	22917452	+	OpenSea	0.79	3.78E−04	0.18	*–*	–	–	–
cg16002891	chr12	6753017	+	N_Shelf	0.80	4.23E−04	0.16	*ACRBP*	Acrosin-binding protein	Body	Acrosomal protein involved in the acrosome formation
cg24365795	chr16	28506015	–	N_Shelf	0.79	4.45E−04	0.16	*APOBR*	Apolipoprotein B receptor	1stExon	Macrophage receptor involved in cholesterol and triglycerides metabolism, and lipid transport
cg10373891	chr13	52338758	+	OpenSea	0.79	4.88E−04	0.11	*–*	–	–	–
cg25946790	chr14	90187489	–	OpenSea	0.79	4.91E−04	0.12	*–*	–	–	–
cg01210113	chr16	11352835	–	S_Shelf	0.79	4.91E−04	0.11	*–*	–	–	–
cg15210829	chr17	2295425	+	N_Shore	0.79	5.01E−04	0.17	*MNT*	Max-binding protein MNT	Body	Binds DNA as a heterodimer with MAX and represses transcription
cg14909856	chr9	117150236	+	OpenSea	0.79	5.20E−04	0.19	*AKNA*	Microtubule organization protein AKNA	5'UTR	Centrosomal protein that plays a key role in cell delamination by regulating microtubule organization; involved in regulation of transcription and inflammatory responses
cg16959766	chr7	36230458	+	OpenSea	0.79	5.21E−04	0.10	*EEPD1*	Endonuclease/exonuclease/phosphatase family domain-containing protein 1	Body	Regulates gene expression linked to cholesterol transport and efflux
cg22461919	chr16	71843295	–	S_Shore	0.76	5.30E−04	0.13	*AP1G1*	AP-1 complex subunit gamma-1	TSS1500	Subunit of clathrin-associated adaptor protein complex 1 that plays a role in protein sorting in the late-Golgi/trans-Golgi network (TGN) and/or endosomes
cg06536724	chr17	64544418	–	OpenSea	0.79	5.31E−04	0.16	*PRKCA*	Protein kinase C alpha type	Body	Calcium-activated serine/threonine-protein kinase involved in apoptosis, cell adhesion, angiogenesis, platelet function and inflammation
cg18169886	chr2	25517869	+	OpenSea	0.79	5.40E−04	0.16	*DNMT3A*	DNA (cytosine-5)-methyltransferase 3A	Body	Required for genome-wide de novo methylation and for the establishment of DNA methylation patterns during development
cg24498454	chr19	48673965	–	S_Shore	0.79	5.43E−04	0.13	*LIG1;C19orf68*	Leucine-rich repeats and immunoglobulin-like domains protein 1	TSS200;1stExon;5'UTR	Feedback negative regulator of signaling by receptor tyrosine kinases
cg19913426	chr17	55213600	–	OpenSea	0.76	5.61E−04	0.17	–	–	–	–
cg06066908	chr6	138044052	+	OpenSea	0.79	5.90E−04	0.15	–	–	–	–
cg18700133	chr17	8013202	–	Island	0.77	6.02E−04	− 0.18	ALOXE3	Hydroperoxide isomerase ALOXE3	Body	Lipoxygenase involved in lipid metabolism (hydroperoxy eicosatetraenoic acid biosynthesis and sphingolipid metabolism)
cg20055664	chr8	134216562	–	OpenSea	0.79	6.37E−04	− 0.10	WISP1	WNT1-inducible-signaling pathway protein 1	Body	Downstream regulator in the Wnt/Frizzled-signaling pathway, associated with cell survival
cg07922719	chr9	117150338	+	OpenSea	0.78	6.55E−04	0.15	AKNA	AT-Hook transcription factor	5'UTR	Centrosomal protein that plays a key role in cell delamination by regulating microtubule organization; involved in regulation of transcription and inflammatory responses
cg26091486	chr20	2687292	+	OpenSea	0.80	6.55E−04	0.12	EBF4	EBF family member 4	Body	Transcriptional factor which recognizes variations of the palindromic sequence 5'-ATTCCCNNGGGAATT-3'
cg26813601	chr15	91105486	+	OpenSea	0.79	7.23E−04	0.18	CRTC3	CREB-regulated transcription coactivator 3	Body	Transcriptional coactivator for CREB1 involved in mitochondrial biogenesis, macrophage activation, lipid catabolism, etc
cg16815249	chr6	111441357	–	OpenSea	0.79	7.46E−04	0.11	SLC16A10	Solute carrier family 16 member 10	Body	Sodium-independent transporter that mediates the uptake of aromatic acids (involved in thiroid hormone metabolism)
cg08609270	chrX	144903125	+	Island	0.76	7.94E−04	− 0.12	SLITRK2	SLIT and NTRK like family member 2	1stExon; 5'UTR	Protein involved in synaptogenesis that promotes excitatory synapse differentiation
cg12078157	chr6	13612218	–	N_Shelf	0.69	7.95E−04	0.13	SIRT5	Sirtuin 5	3'UTR	Mitochondrial NAD-dependent deacylase involved in mitochondrion organization, reactive oxygen species metabolism, etc
cg26360755	chr19	51539314	+	S_Shelf	0.80	8.02E−04	0.10	KLK12	Kallikrein-12	TSS1500	Protein with peptidase activity
cg11913565	chr9	137814810	–	OpenSea	0.80	8.07E−04	0.16	–	–	–	–
cg05968174	chrX	24187388	–	OpenSea	0.78	8.61E−04	0.15	ZFX	Zinc finger X-chromosomal protein	5'UTR	Probable transcriptional activator
cg21110034	chr5	130752683	+	OpenSea	0.79	8.71E−04	0.17	–	–	–	–
cg25550677	chr6	43027568	+	Island	0.80	8.91E−04	0.14	KLC4;MRPL2	Kinesin light chain 4	TSS1500;5'UTR;TSS200	Microtubule-associated force-producing protein that plays a role in organelle transport
cg22348534	chr8	37887424	+	N_Shore	0.79	9.10E−04	0.16	EIF4EBP1	Eukaryotic translation initiation factor 4E-binding protein 1	TSS1500	Repressor of translation initiation that regulates EIF4E activity; regulates protein translation by hormones, growth factors and other stimuli that signal through the MAP kinase and mTORC1 pathways
cg13544012	chr9	135709670	–	OpenSea	0.80	9.10E−04	0.11	C9orf98	Adenylate kinase 8	Body	Nucleoside monophosphate (NMP) kinase that catalyzes the reversible transfer of the terminal phosphate group between nucleoside triphosphates and monophosphates
cg13356427	chr1	6520354	+	N_Shore	0.79	9.26E−04	0.14	ESPN	Espin	3'UTR	Multifunctional actin-bundling protein
cg01515803	chr13	51289817	+	OpenSea	0.79	9.50E−04	0.15	DLEU7	Leukemia-associated protein 7	Body	Protein coding gene deleted In Lymphocytic Leukemia 7

Function of the associated gene is reported as in UniProt database.

**Figure 1:**
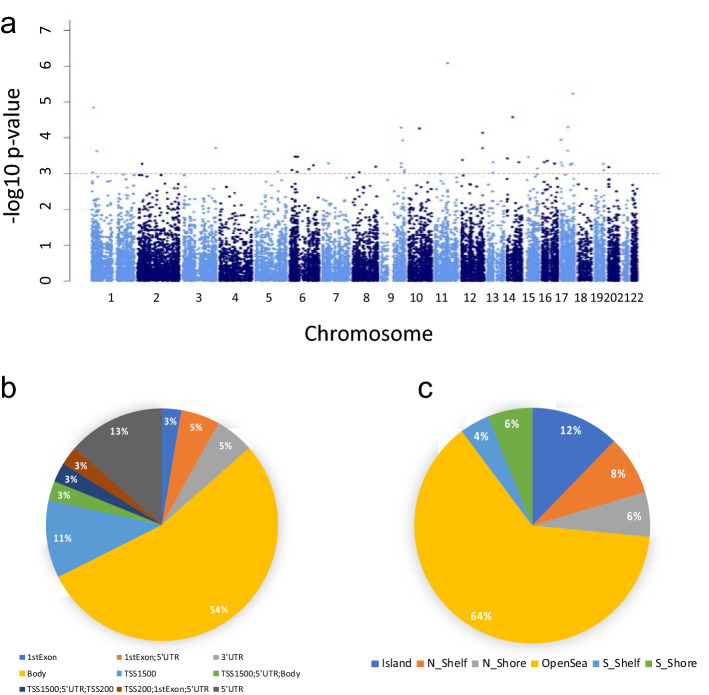
(**a**) Manhattan plot of intervention-sensitive differentially methylated loci (DML). The X-axis represents the chromosomal position and the Y-axis represents the significance on a − log 10 scale. The red and dashed line indicates the threshold for the cut-off p value < 0.001 and DNA methylation mean difference (T2−T1) of 10% or more; (**b**) Percent distribution to standard genomic features of DML with available information (n = 37). 5′UTR = 5′ untranslated region’ 3′UTR = 3′ untranslated region; TSS = transcription start site; TSS200 = 0–200 bp upstream of TSS; 44 TSS1500 = 200–1500 bp upstream of TSS to standard genomic features; (**c**) Percent distribution of intervention-sensitive DML (n = 49) to island relative positions. Shores are considered regions more than 4 kb from CpG islands, shelves are regions 2–4 kb from CpG islands, and other/open sea regions are isolated CpG sites in the genome that do not have a specific designation.

Out of the 49 DML, 87% showed an increase in DNA methylation level from baseline to post-treatment and 37 DML reside in known genes. These 49 DML were distributed across all human chromosomes except the Y chromosome (Fig. 1a) and were most often found within gene bodies (57%), followed by 5′ untranslated regions (27%), and gene promoter regions of genes (up to 1500 basepairs upstream of the gene transcription start site) (16%) (Fig. 1b). Most of the DML were in open sea regions (more than 4 kb from a CpG island) (64%) and 12% were located within CpG islands (Fig. 1c). Considering the probe locations included on the array, the genomic region and location enrichments of the DML were not significant (*p* value > 0.05).

### Functional roles of intervention-sensitive DML

Using a meta-database restricted to the 49 DMLs to identify molecular interactions for network biology (ConsensusPathDB-human tool), we conducted a pathway analysis and found a significant enrichment of functional interactions associated with the nervous, endocrine, immune systems, and processes involved in cancer, diabetes and cardiovascular disease (top 20 pathways with FDR q-value < 0.03, Table 2; all pathways with FDR q-value ≤ 0.05, Supplementary Table S2). These findings support links to neurophysiological processes affected by childhood adversity^3^.

**Table 2:** Top 20 functional interactions of the 49 meditation-sensitive DML (*p* value < 0.001 and mean difference (T2−T1) > 10%) using the ConsensusPath tool.

Consensus Path name	Functional set id	*p* value	FDR q-value
Acetylcholine regulates insulin secretion (Reactome)	118332	0.0002	0.023
Hematopoietic stem cell gene regulation by GABP alpha–beta complex (Wikipathways)	3874547	0.0007	0.023
Amoebiasis—Homo sapiens (human) (KEGG)	167455	0.0010	0.023
Regulation of eif-4e and p70s6 kinase (BioCarta)	282015	0.0012	0.023
Parathyroid hormone synthesis secretion and action—Homo sapiens (human) (KEGG)	167307	0.0013	0.023
Follicle Stimulating Hormone (FSH) signaling pathway (Wikipathways)	3874074	0.0014	0.023
IL8- and CXCR1-mediated signaling events (PID)	264396	0.0015	0.023
GPCR GroupI metabotropic glutamate receptor (INOH)	299561	0.0015	0.023
Thyroid hormone signaling pathway—Homo sapiens (human) (KEGG)	167527	0.0017	0.023
Retinoic acid receptors-mediated signaling (PID)	264415	0.0017	0.023
Alpha 6 Beta 4 signaling pathway (Wikipathways)	3874238	0.0021	0.024
IL8- and CXCR2-mediated signaling events (PID)	264520	0.0022	0.024
African trypanosomiasis—Homo sapiens (human) (KEGG)	167452	0.0024	0.024
Target Of rapamycin (TOR) signaling (Wikipathways)	3873991	0.0025	0.024
PAR1-mediated thrombin signaling events (PID)	264368	0.0037	0.028
PLC beta mediated events (Reactome)	46932	0.0037	0.028
G-protein mediated events (Reactome)	46967	0.0039	0.028
Proton pump inhibitor pathway pharmacodynamics (PharmGKB)	3193117	0.0040	0.028
Endocrine and other factor-regulated calcium reabsorption—Homo sapiens (human) (KEGG)	167435	0.0042	0.028
Regulation of RhoA activity (PID)	264551	0.0044	0.028

Sequence motif enrichments to identify transcription factors binding sites among the 49 intervention-sensitive DMLs revealed 21 significantly enriched motifs (E-value < 0.05, Table 3). The top 5 sequence motifs corresponded to binding sites for ETV4, ZN341, ETV2, SP1, and BC11A transcription factors, which are involved in cell differentiation, regulation of immune homeostasis, blood cell differentiation, immune responses, cancer, cardiovascular disease, diabetes and brain development, respectively, among other biological processes (UniProt database).

**Table 3:** Transcription factor motif enrichment analysis of intervention-sensitive DML.

Rank	Transcription factor	Adj. *P* value (FDR)	*E* value	No of DML	UCSC reference gene name	UniProt function
1	*ETV4*	1.56E−08	6.24E−06	22	ETS translocation variant 4	Transcriptional activator involved in cell differentiation
2	*ZN341*	2.10E−07	8.41E−05	21	Zinc finger protein 341	Transcriptional activator of STAT3 involved in the regulation of immune homeostasis
3	*ETV2*	3.85E−07	1.54E−04	26	ETS translocation variant 2	Transcriptional activator involved in blood cells differentiation, Notch and Wnt signalling pathways
4	*SP1*	1.63E−06	6.53E−04	8	Transcription factor Sp1	Transcritional factor that regulates the expression of genes involved in cell growth, apoptosis, angiogenesis, differentiation and immune responses
5	*BC11A*	2.48E−06	9.96E−04	25	B-cell lymphoma/leukemia 11A	Transcription factor involved in brain development, hematopoiesis, lymphopoiesis
6	*ERG*	2.97E−06	1.19E−03	25	ETS Transcription Factor ERG	Transcriptional regulator involved in cell differentiation
7	*SPI1*	3.39E−06	1.36E−03	16	Transcription factor PU.1	Transcriptional activator involved in the differentiation or activation of macrophages or B-cells
8	*IRF2*	5.87E−06	2.35E−03	12	Interferon regulatory factor 2	Transcriptional activator involved in immune response
9	*ELF3*	7.34E−06	2.94E−03	22	ETS-related transcription factor Elf-3	Transcriptional factor involved in cell differentiation, extracellular matric organization and inflammatory response
10	*SP3*	8.08E−06	3.24E−03	16	Transcription factor Sp3	Transcritional activator of genes involved in cell-cycle regulation, hormone-induction and house-keeping
11	*ETV5*	8.14E−06	3.27E−03	37	ETS translocation variant 5	Transcription factor involved in cell differentiation and cellular response to oxidative stress
12	*IRF8*	1.45E−05	5.83E−03	19	Interferon regulatory factor 8	Transcription negative regulator in cells of the immune system, involved in the immune response
13	*KLF15*	1.48E−05	5.94E−03	15	Krueppel-like factor 15	Transcription factor involved in many processes such as glucose homeostasis, insulin response, Wnt signalling pathway
14	*VEZF1*	2.69E−05	1.08E−02	23	Vascular endothelial zinc finger 1	Transcription factor involved in cellular defense response and angiogenesis
15	*E2F7*	3.69E−05	1.48E−02	17	Transcription factor E2F7	Atypical E2F transcription factor that participates in various processes such as angiogenesis, polyploidization of specialized cells and DNA damage response
16	*SP4*	5.12E−05	2.05E−02	20	Transcription factor Sp4	Transcriptional activator
17	*SPIB*	5.86E−05	2.35E−02	11	Transcription factor Spi-B	Transcriptional activator involved in cell differentiation that can act as a lymphoid-specific enhancer
18	*IRF1*	6.05E−05	2.43E−02	10	Interferon regulatory factor 1	Transcriptional regulator involved in immune response and apoptosis
19	*EHF*	7.73E−05	3.10E-02	22	ETS homologous factor	Transcriptional activator involved in regulating epithelial cell differentiation and proliferation
20	*ZN770*	8.21E−05	3.29E-02	21	Zinc finger protein 770	Transcription regulator
21	*ELF5*	1.04E−04	4.16E-02	21	ETS-related transcription factor Elf-5	Transcriptionally activator involved in cell differentiation, that regulates the later stages of keratinocytes terminal differentiation.

DNA sequences flanking the 49 intervention-sensitive DML (+ /− 250 bp) were used to identify enriched motifs using the AME suite package (*p* value ≤ 0.0001; E-value ≤ 0.05). Transcription factors predicted to bind to each motif, Bonferroni adjusted *p* value, E-value, and the number of DML where the motif is present are shown. Functions of the transcription factors were obtained using the UniProt database.

### Impact of multimodal intervention on epigenetic age acceleration

**Figure 2:**
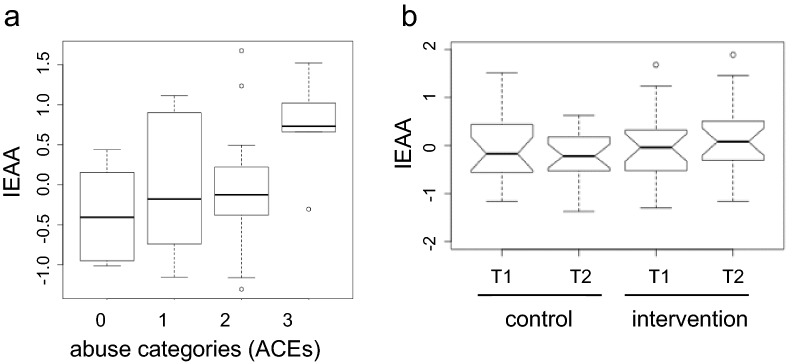
(**a**) Positive correlation between baseline Intrinsic Epigenetic Age Acceleration (IEAA) and exposure to abuse (*p* value = 0.03: r = 0.33). IEAA positive values indicate that biological age is higher than chronological age, whereas negative values indicate that biological age is lower than chronological age. Abuse score was calculated as the sum of individual scores for emotional, physical and sexual abuse on the 10-item ACE scale. (**b**) IEAA adjusted by cell type proportions in control and intervention groups before and after the program. No effect of the intervention on IEAA was detected (Δ IEAA (T2−T1) control vs intervention group, *p* value = 0.23; Supplementary Table S2).

Pearson's correlation analysis revealed no association between baseline Intrinsic Epigenetic Age Acceleration (IEAA) and ACE total score (n = 44; *p* value = 0.43: r = − 0.13). The analyses of the three categories of adversity assessed by the standard ACE questionnaire (i.e. abuse, neglect and household challenges), revealed a weak but significant positive correlation between IEAA and exposure to abuse (emotional, physical and sexual) (*p* value = 0.03: r = 0.33) while neglect (emotional and physical) and household challenges (separation from biological parents, witnessing domestic violence, household substance abuse, mental illness in household and having incarcerated family members) were not associated with epigenetic accelerated aging (neglect: *p* value = 0.07: r = 0.27; household challenges: *p* value = 0.13: r = − 0.23). No significant difference was found in DNA methylation age or Intrinsic Epigenetic Age Acceleration (IEAA) between groups, calculated at T1 and T2 (Fig. 2; Supplementary Table S3a). The intervention did not have any significant impact on the participants’ IEAA according to the ANCOVA model (coefficient = − 0.661, SE = 0.874, *p* value = 0.454) (Supplementary Table S3b).

### Correlation between psychological and DNA methylation outcomes.

Since we previously reported a significant improvement in attention/awareness-related outcomes and a reduction in trauma-related outcomes following the 1-week intervention group program^36^, we next sought to identify DNA methylation changes related to psychological outcomes by comparing differences in DNA methylation levels and changes in the scores for Attention Awareness Scale-Adolescents (MAAS-A), trauma (the Short PTSD Rating Interview (SPRINT)), and the Child PTSD Symptom Scale (CPSS)) at baseline (T1) and post-intervention (T2). This approach revealed significant correlations of DNA methylation levels at 274 CpGs with MAAS-A scores (*p* value < 1 × 10^–3^, r > 0.5, Supplementary Table S4). However, none of these CpGs corresponded to the intervention-sensitive DML described above and they did not show significant functional enrichment (Supplementary Table S5). Improved SPRINT and CPSS scores significantly correlated with DNA methylation levels at 160 and 202 CpGs, respectively (*p* value < 1 × 10^–3^, r > 0.5, Supplementary Tables S6 and S7). Two of these genes corresponded to the intervention-sensitive DMLs described above: *SIRT5* gene (Sirtuin 5; *p* value: 0.0001, r = − 0.59) and *TRAPPC2L* gene (Trafficking Protein Particle Complex Subunit 2L; *p* value: 0.00002, r = − 0.55; Supplementary Table S1. The DNA methylation levels at 35 CpGs correlated with both CPSS and SPRINT scores and Fisher test confirmed that the CpG overlap between scales was significant (*p* value < 1 × 10^–5^). This observation is consistent with the fact that both SPRINT and CPSS scales measure PTSD-related outcomes and that the results from both scales were highly correlated in our previous report (r = 0.833, *p* value < 1 × 10^–3^)^36^. Annotation of these 35 CpGs to genes revealed the known functions of the encoded proteins (Table 4) and an enrichment analysis detected functional interactions involved in metabolic, cardiovascular, immune and neural signaling (*q*-value < 0.04, Supplementary Table S8).

**Table 4:** Function (Uniprot database) of the genes associated to the 35 CpGs found to correlate with both CPSS and SPRINT scales.

CpG ID	Chromosome	Position	Strand	Relative to island position	UCSC reference gene symbol	UCSC reference gene group	UCSC reference gene name	UniProt function
cg11029504	chr9	80512104	+	OpenSea	GNAQ	Body	Guanine nucleotide-binding protein G(q) subunit alpha	Guanine nucleotide-binding protein involved in transmembrane signaling systems, action potential, glutamate signaling pathway, and other processes, as modulator or transducer
cg19041132	chr17	74380824	–	Island	SPHK1	5'UTR; 1stExon; TSS1500	Sphingosine kinase 1	Protein kinase that catalyzes the phosphorylation of sphingosine to form sphingosine 1-phosphate, involved in the regulation of inflammatory response and neuroinflammation
cg07300846	chr16	29888571	+	S_Shore	SEZ6L2	Body	Seizure 6-like protein 2	Protein that contributes to specialized endoplasmic reticulum functions in neurons
cg22531801	chr1	235806070	+	S_Shore	GNG4	5'UTR	Guanine nucleotide-binding protein G(I)/G(S)/G(O) subunit gamma-4	Guanine nucleotide-binding protein involved in transmembrane signaling systems, severla neurotransmitter signaling pathways, and other processes, as modulator or transducer
cg10595547	chr10	119310911	+	N_Shore	–	–	–	–
cg11478273	chr8	128806682	–	Island	PVT1	TSS200	Pvt1 Oncogene	Long non-coding RNA identified as a candidate oncogene
cg01759889	chrX	68725086	+	Island	FAM155B	1stExon; 5'UTR	Family With Sequence Similarity 155 Member B	Component of the NALCN channel complex, involved in the regulation of the resting membrane potential and neuronal excitability
cg25902682	chr5	79461463	+	OpenSea	SERINC5	Body	Serine incorporator 5	Enhances the incorporation of serine into phosphatidylserine and sphingolipids, involved in immunity, lipid metabolism and myelin formation
cg05374956	chr19	5838735	–	OpenSea	FUT6	1stExon; 5'UTR	4-galactosyl-N-acetylglucosaminide 3-alpha-L-fucosyltransferase FUT6	Glycosyltransferase protein involved in glycosylation and lipid metabolism
cg00672930	chr8	130585711	+	OpenSea	CCDC26	Body	Putative coiled-coil domain-containing 26	Long non-coding RNA identified in myelocyte-monocyte lineage
cg12153422	chr14	75075712	+	N_Shore	LTBP2	Body	Latent-transforming growth factor beta-binding protein 2	Plays an integral structural role in elastic-fiber architectural organization and/or assembly
cg05694971	chr15	36872036	–	S_Shore	C15orf41	5'UTR;1stExon	CDAN1-interacting nuclease 1	Involved in erythroid cell differentiation
cg20412539	chr7	999153	–	OpenSea	–	–	–	–
cg17290488	chr5	179281560	–	N_Shelf	C5orf45	Body	MRN complex-interacting protein	Involved in cellular response to DNA damage and the maintenance of genome stability through its association with the MRN damage-sensing complex
cg13451093	chr9	137040612	–	OpenSea	–	–	–	–
cg15757326	chr18	61704584	+	OpenSea	–	–	–	–
cg11856215	chr11	63535358	–	N_Shore	C11orf95	Body	Zinc finger translocation-associated protein	Negative regulator of transcription
cg19816811	chr7	27188364	+	N_Shore	HOXA6	TSS1500	Homeobox protein Hox-A6	Sequence-specific transcription factor which is part of a developmental regulatory system that provides cells with specific positional identities on the anterior–posterior axis
cg00094518	chr7	130418549	+	Island	KLF14	1stExon	Krueppel-like factor 14	Transcription factor involved in various processes including sphingolipid mediated signaling pathway
cg27380803	chr17	62034801	+	OpenSea	SCN4A	Body	Sodium channel protein type 4 subunit alpha	Subunit of a voltage-gated sodium channel complex, involved in neuronal action potential, muscle contraction, etc
cg22246918	chr13	51094655	–	OpenSea	–	–	–	–
cg11336382	chr1	228658646	–	N_Shore	–	–	–	–
cg11283677	chr17	60727886	–	N_Shore	MRC2	Body	C-type mannose receptor 2	May play a role as endocytotic lectin receptor displaying calcium-dependent lectin activity; involved in collagen catabolism, endocytosis, etc
cg07401516	chr5	95571107	+	OpenSea	–	–	–	–
cg24513433	chr18	47088234	–	Island	LIPG	TSS200	Endothelial lipase	Exerts both phospholipase and triglyceride lipase activities; involved in lipid metabolism and cell proliferation
cg20766178	chrX	71131060	+	Island	NHSL2	1stExon	NHS-like protein 2	Protein involed in cell differentiation
cg07724623	chr1	115397409	–	N_Shore	SYCP1	TSS200	Synaptonemal complex protein 1	Major component of the transverse filaments of synaptonemal complexes; involved in cell division
cg22154449	chr18	56930452	+	N_Shore	–	–	–	–
cg16941643	chr9	127277206	–	OpenSea	–	–	–	–
cg23341182	chr10	102046768	+	S_Shore	BLOC1S2	TSS1500	Biogenesis of lysosome-related organelles complex 1 subunit 2	Component of the BLOC-1 complex, involved in biogenesis of lysosome-related organelles, axonal transport, neurite extension, neuron differentiation, and othe processes
cg20655103	chr8	143792280	–	OpenSea	LOC100288181	Body	LncRNA Associated With Ovarian Cancer 1	Long non-coding RNA associated with ovarian cancer
cg19852286	chr5	173237320	+	OpenSea	–	–	–	–
cg06179698	chr2	176671985	–	OpenSea	–	–	–	–
cg06326092	chr16	30034487	–	S_Shore	C16orf92	TSS200	Fertilization-influencing membrane protein	May play a role in sperm-oocyte fusion during fertilization
cg00454932	chr1	171750547	+	Island	METTL13	TSS1500	eEF1A lysine and N-terminal methyltransferase	Methyltransferase involved in the negative regulation of mRNA translation

## Discussion

Here we describe a genome-wide DNA methylation analysis from saliva samples, as an extension of our previous study that showed the mental health benefits of an intensive multimodal 1-week group program involving mindfulness training, artistic expression and EMDR in adolescent girls with a history of 4 or more ACEs (full details on the program protocol and psychological outcomes are described in Roque Lopez et al.^36^).

Forty-nine DML were sensitive to the intervention with a methylation change greater than 10% (*p* value < 0.001). Fifty-four percent of these DML were located in the body of genes, of which 76% showed increases in DNA methylation levels post-intervention, which is generally associated with active transcription in proliferative tissues^37^.

Although DNA methylation analysis from saliva samples might be not representative of other tissue type programming, some reports have shown correlations between DNA methylation levels in brain, blood and saliva^38–41^. A biological pathway-enrichment analysis of the 49 intervention-sensitive DML-associated genes suggests the modulation of several functional processes associated with diseases linked to early childhood adversity, including several biological processes involved in neural signaling and substance abuse disorders (e.g., glutamate receptor, beta agonist/beta blocker, cholinergic, glutamatergic, serotoninergic and dopaminergic synapses and opioid, oxytocin and endocannabinoid signaling, long-term depression and potentiation). These findings are consistent with other reports showing that ACEs can trigger DNA methylation changes in genes that modulate mental health and behavior, such as serotonin transporter and glucocorticoid receptor genes^11,12^, brain-derived neurotrophic factor^14^ and glutamate receptor^15^, oxytocin receptor^12,13^. DML-associated genes also were enriched in processes involved in neural signaling and substance abuse disorders (e.g., glutamate receptor, beta agonist/beta blocker, cholinergic, glutamatergic, serotoninergic and dopaminergic synapses and opioid, oxytocin and endocannabinoid signaling, long-term depression and potentiation). In addition, these DML-associated genes were significantly enriched in processes involved in cardiovascular health (e.g., endothelins, vascular smooth muscle contraction, thromboxane A2 receptor and calcium signaling, beta-agonist/beta-blocker pathways), diabetes (e.g., insulin secretion, leptin signaling, pancreatic secretion, AGE-RAGE signaling) and cancer (e.g., choline metabolism, WNT, ErbB and EGF-EGF receptor signaling, cancer-related microRNAs, NOTCH signaling), which are non-communicable diseases more likely to appear in 18 year old adults or older with a history of at least 4 ACEs than in those with none^3^. Inflammation also has been reported in stress-related disorders^42,43^ and the enrichment analysis suggests that the intervention may regulate inflammation through the modulation of IL8- and chemokine G-coupled receptor CXCR1- and CXCR2-mediated signaling. Furthermore, stress-related DNA methylation changes were associated with the enrichment in several hormone networks (e.g., follicle stimulating hormone signaling, thyroid hormone synthesis and signaling, androgen receptor signaling, aldosterone synthesis and secretion), which are regulated by hypothalamus-pituitary endocrine axes known to be sensitive to stress and childhood adversity^44–47^. Consistent with these findings, the top 5 significantly enriched DNA sequence motifs corresponding to transcription factors binding sites are involved in the regulation of similar processes. ETV4 and ETV2 are transcription factors of the ETS family that have been largely involved in carcinogenesis^48^ and cardiovascular disease^49^. Specificity protein 1 (SP1) is associated with different types of cancer, neurological and cardiovascular disease^50,51^ and ZNF341 is involved in immune-mediated disorders and infection susceptibility by regulating IL-6 signaling^52^. BCL11A is involved in β-hemoglobinopathies, cancer and type II diabetes^53^, neurogenesis^54^ and midbrain dopaminergic neurons^55^.

In our study we found no evidence of association between IEAA and ACE total score, probably because 90% of the participants had a history of 4 or more ACEs. However, our analyses of the three categories of adversity (i.e. abuse, neglect and household challenges), revealed a weak but significant correlation between IEAA and exposure to abuse (emotional, physical and sexual) but not to the other ACE categories. These findings are consistent with data from a prospective study with 974 children showing that girls from age 0–14 years exposed to abuse (i.e., emotional or physical), but not to other individual types of ACEs, presented DNA methylation age acceleration^56^. On the other hand, no effect in the epigenetic aging trajectory was detected in response to the intervention, probably due to its short duration. Future prospective studies including follow-up care and evaluation will be required to explore a putative association of the intervention with changes in the epigenetic aging trajectory in subjects with a history of multiple ACEs.

The DNA methylation changes post-intervention correlated with the CPSS, SPRINT and MAAS-A measured psychological outcomes at 202, 160, and 274 CpGs, respectively. However, only two of these DML, annotated to the *SIRT5* and *TRAPPC2L* genes, showed a change in DNA methylation level greater than 5% (*p* value < 0.001). *SIRT5* (change in DNA methylation = 13%) was associated with CPSS scores and *TRAPPC2L* (change in DNA methylation = 7%) was associated with SPRINT scores. *SIRT5* is a member of the sirtuin family of proteins located predominantly in the mitochondrial matrix, and it protects cells from oxidative stress^57,58^. The effect of traumatic stress on oxidative components and redox-state homeostasis has been documented^59^. These data suggest that the epigenetic modulation of antioxidant-related pathways may be relevant to the psychological benefits of the intervention. SPRINT scores negatively correlated with the DNA methylation levels at the body of *TRAPPC2L* gene, which is involved in intracellular vesicle-mediated transport events^60^ and is functionally associated with neurodevelopmental delay/intellectual disabilities in individuals homozygous for a missense variant^61^.

Taken together, our data support the contribution of epigenetic mechanisms in mediating the effects of the 1-week intervention group program for adolescents exposed to 4 or more ACEs. Future studies are required to examine the functional implications of these changes (i.e., expression levels and activity of candidate genes). The potential relationships of these findings with physiological outcomes may help identify molecular targets aimed to prevent the onset of health disorders and improve the long-term health trajectory in individuals with 4 or more ACEs. Although this level of exposure to adversity increases the risk of adult onset of chronic health problems, behavioral risk, and mortality^3^, ACE screening is not yet integrated into primary care. One of the arguments is the scarce evidence on therapeutic strategies for children or adolescents with a history of multiple victimization^62^. However, the early screening of ACEs is seen by several authors as a promising way to promote child well-being through policy, health education and evidence-based programs for families, children and adolescents^63,64^. Results presented in our previous study^36^, data presented here and recent evidence from other studies^31,32,65,66^ are starting to provide the scientific background to encourage further discussions on future avenues for prevention and treatment of ACEs. Although this study describes a promising short intervention for adolescents with multiple ACEs, the participants may still need group or individual follow-up support in order to enhance and strengthen the benefits from this program. Future prospective studies to assess the stability of the epigenetic changes resulting from the intervention and their potential long-term influence on health are warranted.

## Methods

### Participants

We recruited forty-four adolescent girls, aged 13–16 years, from the foster care system of the Colombian Institute of Family Well-Being (ICBF). All participants were partially or totally separated from their biological families due to inadequate parental care, including abuse and neglect. Exclusion criteria were cognitive impairment, self-harming behavior within the last 6 months, suicidal behavior or ideation or current substance dependence. The flowchart of participants invited, screened, enrolled and completing the study as well as the participants’ demographic information that we could collect have been fully described in our previous report^36^. Participants and their legal representatives provided a written informed consent. We randomized the participants into two groups using a random-number generator. All subjects (intervention and control group; n = 44) underwent parallel and identical assessments at baseline and post-intervention. Participants were informed to which group they had been assigned after the baseline assessment (T1) and subjects assigned to the control group were immediately invited to attend the same program at the end of the study. All the assessments were carried out at the youth care centers from which the participants were recruited.

This research was performed in accordance with relevant guidelines/regulations and in accordance with the Declaration of Helsinki. Informed consent was obtained from all participants and their legal guardians.

### Intervention

The intervention was performed during a school holiday week (June 20–27th, 2019) and it was conducted at a nature retreat facility in Santander, Colombia.

The intervention program included an early morning routine starting with an awakening with soft music and a hot beverage in the garden, followed by a 30 min yoga session^67^ and a guided loving kindness and compassion meditation to cultivate positive affective states^68^. After a healthy breakfast, participants attended a mindfulness practice for adolescents^69^. The program included several sessions per day of artistic expression through art and craft, dramatic play, dance, and music. On days 5 and 6, participants attended two EMDR group protocol sessions/day. During that same week, the control group was engaged in holiday activities proposed by the ICBF. While the intervention and the control group, in their respective locations, engaged in some similar activities (e.g., dance, acting, physical exercise, games, movies), the control group activities did not include approaches to specifically treat traumatic experiences or to promote attentional and emotional regulation. For full details of the intervention program and the control group activities, see Roque López et al.^36^.

### DNA Isolation and Methylation microarray

Before and after the 1-week intervention, saliva samples (1 ml) from all the participants (n = 22/group) were collected using Oragene saliva collection kits and DNA was isolated according to the manufacturer’s protocol. DNA concentration was determined using a Qubit fluorometer (Life Technologies) and normalized to 20 ng/μl for the methylation microarray. Bisulfite conversion was performed with the EZ methylation Gold-kit (cat# D5005, Zymo Research) and the Illumina Infinium MethylationEPIC Beadchip Array was used to quantitatively interrogate at single-nucleotide resolution over 850,000 CpG sites across the genome (Biotech Center, University of Wisconsin-Madison).

### Pre-processing of human MethylationEPIC data

Raw intensity data files were imported into R environment. R package minfi was used to assess sample quality, calculate the detection *p* value of each tested probe, and normalize data^70^. Two samples were discarded as their mean detection *p* value exceeded 0.05. Probes were normal-exponential out-of-band (noob) normalized with dye correction, followed by quantile normalization. No samples showed incorrect sex prediction based on methylation levels. Probes were filtered if at most one sample exhibited a detection *p* value > 0.01, contained a SNP, reported methylation at a SNP, measured methylation at a CH dinucleotide site, had at most one sample with a detection *p* value > 0.01 or were known cross-reactive probes^71,72^. These filtration criteria resulted in 688,000 probes used for further analysis.

Beta values were obtained through minfi and were further converted to M-values for differential analysis.

### Identification of differentially methylated loci (DML)

Linear regression for each tested CpG using an ANCOVA model was employed using R package limma^73^. The treatment effect (difference between the intervention and control group) on DNA methylation level, was estimated using an analysis of covariance (ANCOVA) of the outcome (T2) with the baseline (T1) as covariate. BMI, age, ACE score and cell type proportions (surrogate variables) were also included as covariates. In this model, the mean posttest difference between the groups is used to estimate the outcome (DNAm T2 ~ Group (control/int) + age + bmi + ACE + DNAm T1 + surrogate variables)^74^. Surrogate variables were assessed by R package sva^75^, which identified a total of 3 variables. For quality control purposes cell type proportions were also calculated using R package RefFreeEWAS. The correlation of *p* values between these two approaches was 0.86, indicating the accuracy of both measures. *P* values corrected and uncorrected by FDR were obtained. To assess systematic bias of the linear regression model, the genomic inflation factor was calculated for the obtained *p* values, yielding a genomic inflation factor of ~ 1, suggesting no bias in these methods. In our study, the relatively small sample size, together with some characteristics inherent to the array (measure of continuous variables in large cell numbers, non-variability of many sites on the array, correlation between neighboring probes on the array) likely resulted in the absence of FDR adjusted DML^76,77^. However, an FDR adjustment assumes independence in the comparisons, and DNA methylation levels across the genome are not independent. Thus, several studies have taken an approach that requires a larger effect size (i.e., > 10%) with a more liberal *p* value cut-off^78–81^. Therefore, to detect intervention-sensitive DML, we established as cut-off a *p* value ≤ 0.001 combined with an average difference in methylation between T1 and T2 greater than 10%.

### Functional analysis

Gene ontological enrichment of biological processes were identified using the ConsensusPathDB-human database as implemented in the Functional Enrichment module of the EASIER R package^82^. This database integrates interaction networks in Homo sapiens including metabolic, biochemical and gene regulatory signaling and drug-target interactions. FDR-corrected *p* values < 0.05 were considered significant.

The DNA sequences flanking the DML of (+ /− 250 nucleotides) were used to find enriched motifs using the AME suite package (MEME Suite online platform)^83^. An E-value cut-off of 0.05 was established to identify significantly enriched motifs, as recommended by MEME developers^83^.

### Estimation of the impact of the multimodal intervention on epigenetic age acceleration

We explored the associations between Intrinsic Epigenetic Age Acceleration (IEAA) and ACE scores using the basal DNA methylation data from both groups and the ACE scores that we previously described in the same sample^36^. Child epigenetic age based on the Pediatric-Buccal-Epigenetics’ (PedBE) clock^21^ was calculated using the methylclock R package^84^. The package provides the following parameters: (i) DNA methylation predicted age (biological age) in years, (ii) age acceleration, difference between DNAm and chronological age in years; (iii) Intrinsic Epigenetic Age Acceleration (IEAA), obtained after regressing chronological age and cell type proportions on biological age. Pearson's correlation analysis was used to explore associations between basal Intrinsic Epigenetic Age Acceleration (IEAA) adjusted by cell type proportions, ACE total score and the number of ACEs from each one of the three categories of adversity (i.e. abuse: emotional, physical and sexual; neglect: emotional and physical; household dysfunction: separation from biological parents, witnessing domestic violence, household substance abuse, mental illness in household and having incarcerated family members), assessed by the 10-item ACE questionnaire derived from the Kaiser Permanente ACEs Study^85^ (full details on frequency and patterns of ACEs in this sample are described in Roque Lopez et al.^36^). We used an ANCOVA model (see “Methods”) to assess the potential impact of the intervention on IEAA. This model included group (intervention or control) as the independent variable, IEAA at T2 from both groups as the dependent variable, and it was adjusted by basal IEAA (T1), BMI, and ACE score, considering *p* values < 0.05 as significant.

### Correlation between psychological phenotypic measures and DNA methylation

PTSD and awareness and attention-related outcomes of this intervention in this same sample were assessed by SPRINT, CPSS and MAAS-A scales and are fully reported in our previous report^36^. Here we conducted correlations between changes in the above-mentioned scales and changes in DNA methylation (T2−T1) of each CpG.

Linear regression for each tested CpG using an ANCOVA model was employed using R package limma^73^. Separate models for each psychological scale were constructed, controlling for age, BMI and ACES score. Surrogate variables were assessed by the R package sva^75^*.* To assess systematic bias of the linear regression model, the genomic inflation factor was calculated for the obtained *p* values, yielding a genomic inflation factor of ~ 1, suggesting no bias. Pearson’s correlation coefficients (r) were calculated for continuous variables of interest with beta-values. Correlations with an uncorrected *p* value < 1 × 10^–3^, and a correlation coefficient r > 0.5 were considered significant for the current study.

## Supplementary Information

Supplementary Information.

## Data Availability

The datasets generated during and/or analysed during the current study are available from the corresponding author on reasonable request.
